# Advances in MERS-CoV Vaccines and Therapeutics Based on the Receptor-Binding Domain

**DOI:** 10.3390/v11010060

**Published:** 2019-01-14

**Authors:** Yusen Zhou, Yang Yang, Jingwei Huang, Shibo Jiang, Lanying Du

**Affiliations:** 1 State Key Laboratory of Pathogen and Biosecurity, Beijing Institute of Microbiology and Epidemiology, Beijing 100071, China; yszhou@bmi.ac.cn; 2 Institute of Medical and Pharmaceutical Sciences, Zhengzhou University, Zhengzhou 450052, China; 3 Department of Molecular Biophysics and Biochemistry, Yale University, New Haven, CT 06520, USA; y.yang@yale.edu; 4 Lindsley F. Kimball Research Institute, New York Blood Center, New York, NY 10065, USA; JHuang2@nybc.org (J.H.); SJiang@nybc.org (S.J.)

**Keywords:** Coronavirus, MERS-CoV, spike protein, receptor-binding domain, vaccines, therapeutics

## Abstract

Middle East respiratory syndrome (MERS) coronavirus (MERS-CoV) is an infectious virus that was first reported in 2012. The MERS-CoV genome encodes four major structural proteins, among which the spike (S) protein has a key role in viral infection and pathogenesis. The receptor-binding domain (RBD) of the S protein contains a critical neutralizing domain and is an important target for development of MERS vaccines and therapeutics. In this review, we describe the relevant features of the MERS-CoV S-protein RBD, summarize recent advances in the development of MERS-CoV RBD-based vaccines and therapeutic antibodies, and illustrate potential challenges and strategies to further improve their efficacy.

## 1. Introduction

Middle East respiratory syndrome (MERS) coronavirus (CoV) is an infectious virus that was first reported in June 2012 [1]. MERS-CoV may infect people of any age, but older age, underlying comorbidity (such as diabetes mellitus, renal disease, respiratory disease, heart disease, and hypertension), and delayed confirmation or late diagnosis are all factors that affect MERS disease outcomes and mortality [2,3,4,5,6,7]. Sex could be a factor in MERS epidemiology, as more males seem to be affected than females [8,9,10]. MERS-CoV infection of women during pregnancy has adverse outcomes, with fetal mortality of ~27%; however, only a limited number of pediatric MERS-CoV infections occur [11,12,13,14]. At the end of December 2018, 2,279 laboratory-confirmed MERS infections were reported globally (in 27 countries), leading to 806 deaths, and a mortality of 35.3%. Among these infections, 1,901 (83.4%) were reported in Saudi Arabia, with mortality in 732 individuals (38.5%) (http://www.emro.who.int/health-topics/mers-cov/mers-outbreaks.html). The largest MERS outbreak outside Saudi Arabia occurred in South Korea in 2015, with 186 cases and 38 deaths [9,15,16]. The most recent MERS cases were reported in 2018 in South Korea, the United Kingdom, and Malaysia, in addition to Saudi Arabia, the United Arab Emirates, and Oman (http://www.who.int/emergencies/mers-cov/en/).

MERS-CoV is thought to have originated in bats [17,18,19,20]. MERS-like viruses have been isolated from bats that use (at lower efficiency) the same receptor for cell entry as the MERS-CoV isolated from humans [21,22,23]. Dromedary camels are potential intermediates for long-term evolution of MERS-CoV and seasonal zoonotic transfer of virus to humans [24,25,26,27]. Antibodies specific to MERS-CoV, particularly neutralizing antibodies that neutralize MERS-CoV infection, have been detected in the sera of dromedary camels from a number of countries and regions, with high positive detection rates [28,29,30,31,32,33,34]. In addition to camel-to-camel transmission of MERS-CoV, camel-to-human transmission can occur via direct or indirect contact with sick animals [35]. There is a high prevalence of MERS-CoV infection in camel workers, particularly in Saudi Arabia [27]. Following infection of individuals, human-to-human transmission of MERS-CoV occurs frequently, and mainly contributes to community or healthcare-associated outbreaks [36,37,38,39,40,41,42,43,44,45]. Infection of humans by MERS-CoV, which mainly occurs through the lower respiratory tract, causes severe respiratory symptoms, leading to failure of the respiratory system and/or other organs [7,46,47]. On rare occasions, the human intestinal tract can be an alternative infection route [46,48].

Anti-MERS-CoV antibodies, including neutralizing antibodies, are found in infected patients, some of whom have persistently presented such antibodies for up to 34 months after the disease outbreak [49,50]. Antibody responses, and particularly neutralizing antibody responses, are crucial factors in the successful treatment of MERS-CoV infections in humans [50,51,52]. In this regard, the plasma of convalescent MERS-CoV-infected patients is useful for treatment of MERS-CoV infection clinically, but it requires a neutralizing antibody titer (e.g., 50% plaque-reduction neutralization titer: PRNT_50_) ≥1:80 to obtain effective therapeutic results [51,52,53]. Currently, a human polyclonal IgG antibody (SAB-301) that is produced by transchromosomic cattle immunized with a MERS-CoV vaccine [54], as well as several vaccine candidates (https://clinicaltrials.gov/ct2/show/NCT03399578; https://clinicaltrials.gov/ct2/show/NCT03615911) [55], have been tested in human clinical trials. However, as yet no vaccines or therapeutic agents have been approved for the prevention or treatment of MERS, indicating the need for further development of novel and effective vaccines and therapeutics against MERS-CoV infection.

In the following review, we will briefly describe MERS-CoV spike (S) protein receptor-binding domain (RBD), and summarize recent advances in the development of RBD-based MERS-CoV vaccines and therapeutics, as well as the potential challenges and future expectations for their successful development.

## 2. MERS-CoV S Protein RBD

**Figure 1:**
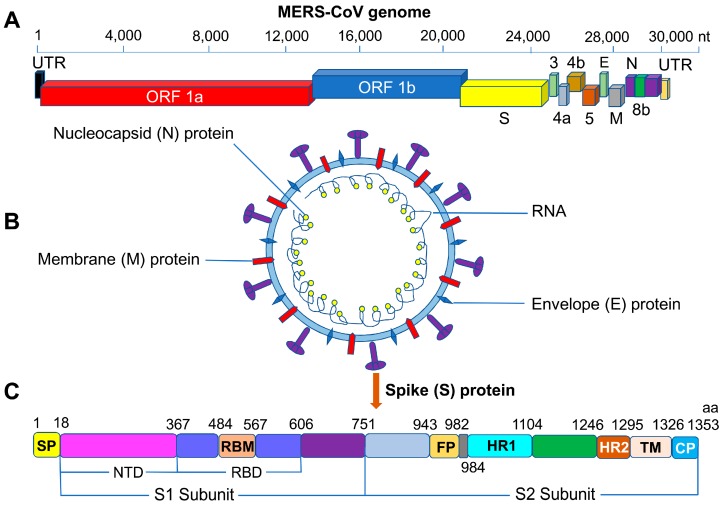
Schematic structures of MERS-CoV S protein. (**A**) MERS-CoV genomic structure, with the untranslated region (UTR), open reading frame regions ORF1a and ORF1b, spike (S), envelope (E), membrane (M), and nucleocapsid (N) genes. (**B**) Schematic structure of the MERS-CoV virion and its major structural proteins. (**C**) Schematic structure of the MERS-CoV S protein and its functional domains, including the N-terminal domain (NTD), receptor-binding domain (RBD), receptor-binding motif (RBM), fusion peptide (FP), heptad repeat region 1 and 2 (HR1 and HR2), transmembrane region (TM), and cytoplasmic tail (CP). aa, amino acid; MERS-CoV, Middle East respiratory syndrome coronavirus; nt, nucleotide.

MERS-CoV belongs to the genus *Betacoronavirus*, as does the severe acute respiratory syndrome (SARS)-CoV [56,57]. MERS-CoV is a positive-sense, single-stranded RNA virus with a genome that encodes the structural proteins S, envelope (E), membrane (M), and nucleocapsid (N), as well as non-structural proteins (nsp1–16) translated from open reading frames (ORFs) 1a and 1b, and several accessory proteins, such as 3, 4a, 4b, 5, and 8b (Figure 1A,B) [58,59,60,61]. The functions of these proteins have been elucidated. As an example, nsp1 is known to have an endonucleolytic RNA-cleavage function in the promotion of infectious virus particle production in human cells [62], and it also participates in efficient viral replication through interaction with viral RNA, probably via a cis-acting element at the 5′-terminal coding region [63]. Evidence indicates that nsp16 is necessary for interferon resistance and viral pathogenesis [64], whereas nsp15 is a nidoviral uridylate-specific endoribonuclease, the structure of which has recently been resolved, and its activity may be mediated by catalytic residues, oligomeric assembly, or RNA-binding efficiency [65]. In addition, 4a protein recognizes and binds double-stranded (ds)RNA, in which four residues of the protein might be crucial for 4a-dsRNA stability [66], and it also inhibits protein kinase R-mediated antiviral stress responses [67]. 4a mediated-inhibition of stress-granule formation may facilitate viral translation, resulting in efficient MERS-CoV replication [68]. The MERS-CoV 4b protein interferes with the NF-*κ*B-dependent innate immune response during infection [69].

MERS-CoV S protein has an important role in viral pathogenesis, determining host tropism and entry into host cells [58,70,71]. The S protein contains an S1 subunit at the N terminus and an S2 subunit at the C terminus. The S1 subunit is composed of the N-terminal domain (NTD) and RBD [58,72,73]. The RBD has a key role in the mediation of binding of MERS-CoV to cells expressing dipeptidyl peptidase 4 (DPP4) receptor, enabling the virus to enter into target cells by fusing with cell membranes through the formation of a fusion core (Figure 1C) [74,75,76,77]. The S protein requires host cellular proteases for its activity in viral entry, but although evidence initially indicated that cellular furin activates S protein, subsequent results have demonstrated no evidence for the involvement of furin during viral entry [71,78]. The DPP4 receptor varies among different host species, and MERS-CoV is thought to use multiple pathways to enable rapid adaptation to species-specific variations [79,80,81].

In addition to DPP4, MERS-CoV can bind to sialic acid via the S1 subunit of S protein, or utilize the membrane-associated 78 kDa glucose-regulated protein (GRP78) to attach to target cells, suggesting that these proteins may also have roles in virion attachment [82,83].

**Figure 2:**
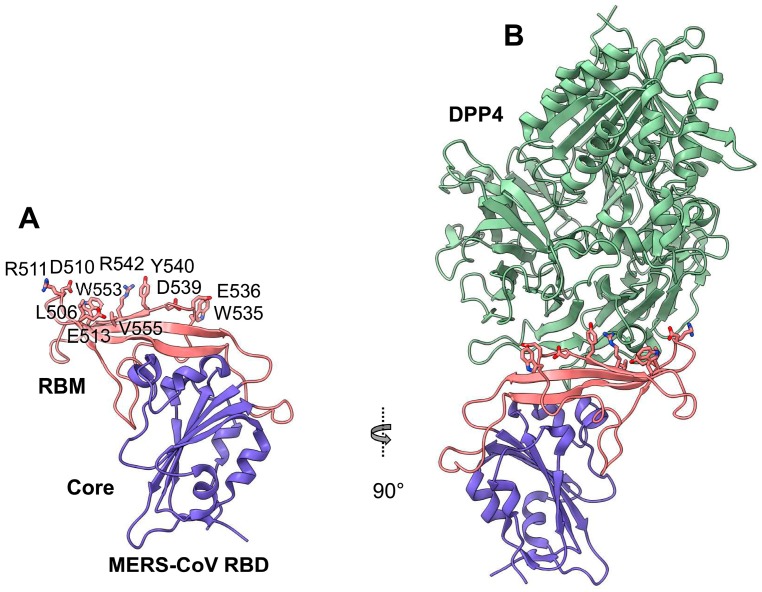
Structural basis of MERS-CoV S-protein RBD–DPP4 interaction. Structural data for the complex of MERS-CoV S-protein RBD bound to DPP4 are from the protein data bank (PDB) (ID: 4KR0). The MERS-CoV RBD core is colored in blue, the RBM is colored in red, and DPP4 is colored in green. The RBM residues directly involved in DPP4 binding are shown as sticks. DPP4, dipeptidyl peptidase 4; RBD, receptor-binding domain; RBM, receptor-binding motif; S, spike protein.

**Figure 3:**
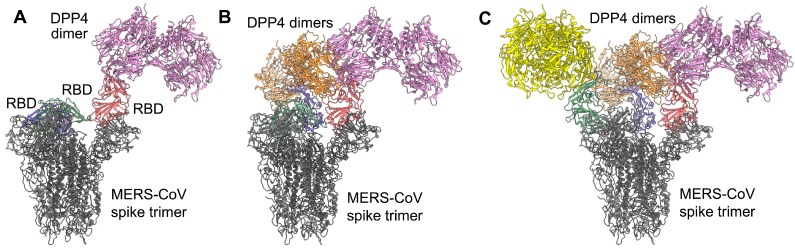
Models of the MERS-CoV S-protein trimer bound to DPP4. The models were generated by superimposing the MERS-CoV RBD in the structure of the MERS-CoV S-protein RBD–DPP4 complex (PDB ID: 4KR0) onto the RBDs in the structures of MERS-CoV S-protein trimers with (**A**) one RBD (PDB ID: 5X5F), (**B**) two RBDs (PDB ID: 5X5C), or (**C**) three RBDs (PDB ID: 5X59) in the “standing” conformation. The MERS-CoV S-protein trimers are colored in gray, with three RBDs colored in red, blue, and green. Three DPP4 dimers are colored in plum, orange, and yellow. DPP4, dipeptidyl peptidase 4; PDB, protein data bank; RBD, receptor-binding domain; S, spike protein.

The structures of MERS-CoV RBD alone and complexed with DPP4 have been determined (Figure 2) [77,84,85]. The RBD has a fold-rich tertiary structure, which consists of a core and a receptor-binding motif (RBM), with stabilization provided by four disulfide bonds and two glycans [77]. A number of RBD residues are located at the DPP4-binding interface, and they have a critical role in RBD–DPP4 binding [77,84,85]. Structural analysis of MERS-CoV trimeric S protein has identified specific features of the RBD and its complex with DPP4. Notably, in the prefusion conformation of the S trimer, individual RBDs are either buried (lying state) or exposed (standing state), and this flexibility presumably facilitates recognition by DPP4 [86]. Other structural studies have revealed four S-trimer conformational states, in which each RBD is either tightly packed at the membrane-distal apex or rotated into a receptor-accessible conformation, suggesting fusion initiation through sequential RBD events [87]. In configurations with one, two, or three RBDs rotated out, RBD determinants are exposed at the apex of the RBD–DPP4 complex, and they are accessible for interaction with DPP4 (Figure 3) [87].

The function and structure of the S-protein RBD demonstrate that it is an important target for development of vaccines and therapeutic agents against MERS-CoV.

## 3. Recent Advances in the Development of Vaccines Based on the MERS-CoV S-Protein RBD

A number of MERS vaccines have been developed based on viral RBD, including nanoparticles, virus-like particles (VLPs), and recombinant proteins, and their protective efficacy has been evaluated in animal models, including mice with adenovirus 5 (Ad5)-directed expression of human DPP4 (Ad5/hDPP4), hDPP4-transgenic (hDPP4-Tg) mice, and non-human primates (NHPs) [88,89,90,91,92,93,94]. Features of these RBD-based vaccines, in terms of functionality, antigenicity, immunogenicity, and protective ability, are shown in Table 1. 

A soluble nanoparticle vaccine formed in *Escherichia coli* by the RNA-mediated folding of a RBD-ferritin (FR) hybrid elicits robust RBD-specific antibody and cellular immune responses in mice, producing antisera that effectively block the binding of RBD to hDPP4 in vitro [89]. The adjuvants alum and the squalene-based MF59 significantly augment the antibody titers and T-cell responses induced by RBD–FR nanoparticle vaccines engineered with or without a SSG linker [89]. Similarly, a chimeric, spherical VLP (sVLP) vaccine expressing MERS-CoV RBD induces specific antibody and cellular immune responses in mice, preventing pseudotyped MERS-CoV entry into susceptible cells [90]. The protective efficacy of these two types of MERS vaccine does not yet seem to have been investigated in a viral-challenge animal model.

Recombinant vaccines involving RBD subunits have been extensively studied for protection against MERS-CoV infection in MERS-CoV-susceptible animal models [93,95,96,97,100,101]. A recombinant RBD (rRBD) fragment (residues 367–606) expressed in insect cells elicits an antibody response and the production of neutralizing antibodies in mice and NHPs [88,91]. It gives incomplete protection in MERS-CoV-challenged NHPs, with the alleviation of pneumonia and clinical manifestations, as well as the reduction of viral load in lung, trachea, and oropharyngeal swabs [91].

A MERS-CoV S-protein RBD fragment containing residues 377–588 has been identified as a critical neutralizing domain [95]. A treatment regimen involving two doses of a fusion of this fragment and the Fc region of human IgG (S377-588-Fc) four weeks apart is able to induce strong, long-term antibody responses (including production of neutralizing antibodies) in mice [98]. These responses are significantly greater than those with a single dose or two doses at intervals of one, two, or three weeks [98]. rRBDs with single or multiple mutations corresponding to S-protein sequences of MERS-CoV strains isolated from humans or camels from 2012 to 2015 have also been studied [100]. All these rRBDs bind RBD-specific neutralizing monoclonal antibodies (mAbs) and DPP4, and are highly immunogenic, eliciting the production of S1-specific antibodies in mice, which cross-neutralizes multiple MERS pseudoviruses and live MERS-CoV [100]. A trimeric RBD-Fd protein formed by fusing a MERS-CoV RBD fragment (residues 377–588) to the foldon trimerization motif, binds strongly to DPP4, and elicits robust and long-term responses with the production of MERS-CoV S1-specific antibodies and neutralizing antibodies in mice, and protects hDPP4-Tg mice against MERS-CoV infection [94].

The protection provided by existing subunit vaccines based on wild-type MERS-CoV RBD is not complete, with survival rates in hDPP4-Tg mice after a MERS-CoV challenge of ~67% for S377-588-Fc and 83% for RBD-Fd [94,98]. However, a variant RBD (T579N) vaccine produced by masking a non-neutralizing epitope at residue 579 with a glycan probe has both functionality in binding DPP4, and antigenicity in binding four potent MERS-CoV RBD-specific neutralizing mAbs (hHS-1, m336, m337, and m338) [93]. The T579N vaccine has significantly greater efficacy than the wild-type RBD vaccine, and it fully protects against a lethal MERS-CoV challenge in immunized hDPP4-Tg mice [93], demonstrating the possibility of developing RBD-based MERS-CoV vaccines with high efficacy.

## 4. Recent Advances in the Development of Therapeutics Based on the MERS-CoV S-Protein RBD

MERS-CoV RBD-targeting antibodies have been developed as effective tools to prevent and treat MERS-CoV infections [102,103,104,105,106,107,108,109]. These antibodies generally have greater neutralizing activity against MERS-CoV infection than non-RBD S1-based or S2-based antibodies [58,103,110,111]. The prophylactic and therapeutic efficacies of RBD-targeting antibodies have been tested in Ad5/hDPP4 mice, hDPP4-Tg mice, and NHPs [102,104,112,113,114].

In an earlier review, we described the antiviral mechanisms, in vivo protection, and crystal structures of previously reported MERS-CoV RBD-specific mAbs, including mouse mAbs Mersmab1, 2E6, 4C2, F11, and D12, and human mAbs LCA60, MERS-4, MERS-27, REGN3048, REGN3051, 1E9, 1F8, 3A1, 3B11, 3B12, 3B11-N, 3C12, M14D3, m336, m337, m338, hMS-1, and 4C2h [58]. In this review, we focus on newly reported antibodies targeting MERS-CoV S-protein RBD, or on newly identified features of existing mAbs that were not described previously (Table 2) [102,112,113,114,115].

### 4.1. MERS-CoV S-Protein RBD-Targeting mAbs

**Figure 4:**
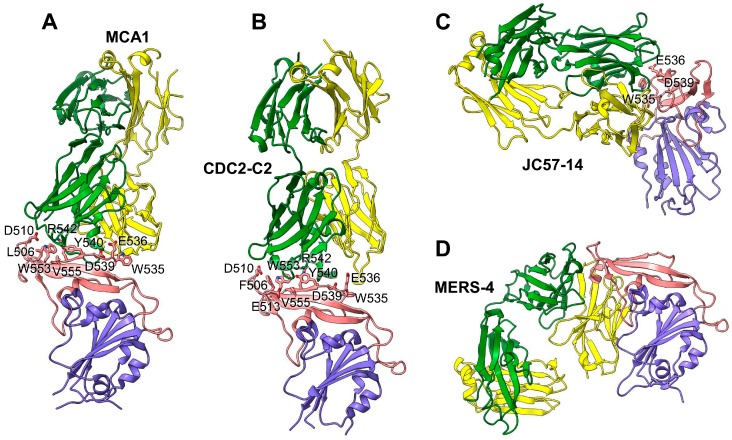
Structural basis for MERS-CoV RBD recognition by neutralizing mAbs. (**A**) Crystal structure of MERS-CoV RBD complexed with MCA1 mAb (PDB ID: 5GMQ). (**B**) Crystal structure of the MERS-CoV RBD complexed with CDC2-C2 mAb (PDB ID: 6C6Z). (**C**) Crystal structure of the MERS-CoV RBD complexed with JC57-14 mAb (PDB ID: 6C6Y). (**D**) Crystal structure of MERS-CoV RBD complexed with MERS-4 mAb (PDB ID: 5ZXV). The MERS-CoV RBD core is colored in blue, the RBM is colored in red, and the heavy chains and light chains of each mAb are colored in green and yellow, respectively. The DPP4-binding residues that are blocked by each mAb are shown as sticks. mAb, monoclonal antibody; MERS-CoV, Middle East respiratory syndrome coronavirus; PDB, protein data bank; RBD, receptor-binding domain; RBM, receptor-binding motif.

RBD-targeting human mAbs have been extensively reported. Most of these mAbs can neutralize pseudotyped or live MERS-CoV in vitro, and some have shown protection against MERS-CoV infection in animal models in vivo [102,112,113,114,115]. The structures of several of these mAbs with their antigen-binding fragments (Fabs) or single-chain variable fragments (scFvs) complexed with RBD are known (Figure 4) [102,112,113,114,115]. Binding of these mAbs to RBD involves two major recognition modes, with binding to RBD residues contacted by or overlapping with DPP4 (as is the case for GD-27, MCA1, and CDC2-C2), or with binding to the RBD residues outside of the DPP4-binding interface (as seen with MERS-4) (Table 2). 

The human mAbs MERS-GD27 and MERS-GD33 each recognize distinct regions of the RBD [113]. These mAbs have a synergistic effect in the neutralization of pseudotyped MERS-CoV in vitro, with a much lower half-maximal inhibitory concentration (IC_50_) for their use in combination than separately [113]. An analysis of crystal structures has indicated that MERS-GD27 binds RBD at the DPP4-binding site, and that the neutralization and recognition epitopes almost completely overlap this site, as seen previously for MERS-CoV RBD-targeting neutralizing mAbs, such as m336 [106,113]. The MERS-GD27 mAb protects hDPP4-Tg mice from MERS-CoV challenge, both preventively and therapeutically, with significantly lower lung virus titers and RNA copy numbers at day 5 post-challenge, and higher survival rates (60% for pre-challenge vaccination and 40% for post-challenge vaccination) relative to control mice treated with an irrelevant mAb [112]. 

The human mAb MCA1 was isolated from a MERS survivor via the construction of a phage-display antibody library from peripheral B cells [114]. Crystal structure analysis indicates that MCA1 binds MERS-CoV S-protein RBD at residues involved in receptor binding, thus interfering with RBD binding to hDPP4 (Figure 4A) [114]. This mAb prophylactically and therapeutically inhibits MERS-CoV replication in common marmosets, resulting in significantly improved outcomes and reduced lung disease, compared with unvaccinated controls, and undetectable virus titers 3 days post-challenge [114].

A probe-based single-B-cell cloning strategy has been used for the isolation of CDC2-C2 and CDC2-C5 mAbs from a patient convalescing from MERS, as well as for the isolation of JC57-11 and JC57-14 mAbs from NHPs immunized with MERS-CoV full-length S DNA and protein [102]. All these antibodies have neutralizing activities against both pseudotyped and live MERS-CoV. Among them, CDC2-C2 is the most potent against 10 pseudotyped MERS-CoV strains, with neutralization IC_50_ values ranging from 0.002 µg/mL to 0.011 µg/mL [102]. Crystal-structure analysis of the CDC2-C2 and JC57-14 Fab–RBD complexes indicates that both mAbs bind RBD in the “out” (exposed) position, with the CDC2-C2 RBD binding overlapping with the DPP4-contacting residues (Figure 4B,C) [102]. In addition, CDC2-C2 prophylactically protects hDPP4-Tg mice from MERS-CoV infection, resulting in no detectable viral replication in the lungs three days post-challenge, and no fatalities over 28 days of observation [102].

The human mAb MERS-4 also neutralizes pseudotyped MERS-CoV and, notably, displays synergistic neutralization in combination with the MERS-CoV S-protein RBD-targeting MERS-27 and m336 mAbs [106,118], as well as the S-protein NTD-targeting 5F9 mAb, in each case with dramatic reduction of the IC_50_ compared with individual mAbs [115]. Structural analysis of a MERS-4-Fab–RBD complex revealed that MERS-4 binds the RBD from outside the DPP4-binding interface, rather than competing with DPP4 (Figure 4D). Unlike MERS-27, which binds RBD regardless of its conformational state within the S trimer, MERS-4 binds RBD in the “standing” position where its epitopes are readily exposed and accessible [115]. Thus, MERS-4 displays unique epitope specificity, and an unusual mechanism of action involving indirect interference with DPP4 binding through conformational changes, which may explain the observation of synergistic neutralization in combination with other mAbs [115].

### 4.2. Nanobodies Targeting the MERS-CoV S-protein RBD

Single-domain antibody fragments (VHHs), or nanobodies, are the antigen-recognition regions of camelid heavy-chain-only antibodies (HcAbs), which do not contain light chains. VHHs are easily expressed with high yield, and they have intrinsic stability, strong binding affinity, and specificity to target antigens, and they have therefore been developed as important therapeutic tools against viral infection, including that of MERS-CoV [116,117,119,120,121,122,123].

Four VHHs (VHH-1, VHH-4, VHH-83, and VHH-101) have been identified from bone marrow cells of dromedary camels immunized with modified vaccinia virus (MVA) expressing MERS-CoV S protein, and challenged with MERS-CoV [116]. These VHHs bind MERS-CoV S protein with low *K*_d_ values (0.1–1 nM), recognize an epitope at residue D539 of RBD, and neutralize MERS-CoV (PRNT_50_, 0.0014–0.012 µg/mL) [116]. These four monomeric VHHs have each been fused with a C-terminal human IgG2 tag to generate four HCAbs (HCAb-1, HCAb-4, HCAb-83, and HCAb-101), with a higher binding affinity and a longer half-life than the free VHHs [116]. Studies of protective efficacy show that hDPP4-Tg mice (K18) injected with monomeric VHH-83 (20 or 200 µg per mouse) lose weight, and die within seven days post-infection, possibly because of the short half-life of the VHH. However, when the mice are injected with HCAb-83 (200 µg per mouse), which has an extended half-life (~4.5 days), protection against MERS-CoV is complete, with no viral titers or pathological changes in the lungs of virally challenged mice [116].

By immunizing llamas with a recombinant RBD fragment (residues 377–588) fused to a C-terminal human IgG Fc tag (S377-588-Fc), we constructed a VHH library, and we used it to generate a monomeric VHH, NbMS10, and a human Fc-fused VHH, NbMS10-Fc [117]. Both VHHs can be expressed in a yeast expression system to high purity, and bind RBD with high affinity, recognizing a conformational epitope (residue 539) at the RBD–DPP4 interface, and blocking the binding of RBD to DPP4. These VHHs, particularly NbMS10-Fc, potently cross-neutralize pseudotyped MERS-CoV strains isolated from different countries, hosts, and time periods [117]. Importantly, the Fc-fused NbMS10-Fc significantly improves the serum half-life of NbMS10, and a single-dose treatment of hDPP4-Tg mice with this agent completely protects them against lethal MERS-CoV challenge [117]. These single-domain VHHs demonstrate the feasibility of developing cost-effective, potent, and broad-spectrum therapeutic antibodies against MERS-CoV infection.

## 5. Potential Challenges and Future Perspectives

Compared with vaccines based on MERS-CoV full-length S protein, which have the potential to attenuate neutralizing activity or enhance immune pathology, vaccines developed from MERS-CoV S-protein RBD are safer, and they do not cause immunological toxicity or eosinophilic immune enhancement [55,99,110,124]. Moreover, RBD-based therapeutic antibodies are generally more potent than non-RBD S1-based or S2-based antibodies [58,104,111]. Hence, RBD-based vaccines and therapeutic antibodies have the potential for further development as effective tools to prevent and treat MERS-CoV infection.

Despite their acknowledged advantages, there are some issues associated with RBD-based interventions that need to be addressed. For example, RBD is under a high level of pressure of positive selection, and mutations occur in the RBD-DPP4 binding interface that might reduce the efficacy of these treatments [100,125,126,127]. One possible way to avoid this effect, and to delay the emergence of escape mutants is to combine RBD-targeting therapeutics with those targeting other regions of the S protein, or to combine antibodies recognizing distinct epitopes within the RBD [102,128]. Such combinatorial strategies could also dramatically reduce antibody neutralization doses, providing feasible means to combat the continual threat of MERS-CoV.

Some recent advances have been made in the structure-guided design of anti-MERS-CoV interventions. Structurally designed inhibitors of the 3CL protease have demonstrated potency against MERS-CoV [129]. Also, a structurally designed S-protein trimer in the optimal prefusion conformation is shown to elicit production of high titers of anti-MERS-CoV neutralizing antibodies [87]. Indeed, based on the previous studies on the structural design of MERS-CoV RBD, non-neutralizing epitopes in the RBD can be masked, to refocus the immunogenicity of the RBD on the neutralizing epitopes, and thus to enhance its ability to confer immune protection [93]. Results from these structure-based studies will help to inform the design of innovative RBD-based anti-MERS-CoV vaccines and therapeutics with improved efficacy.

## Figures and Tables

**Table 1:** Vaccines based on MERS-CoV RBD. Live MERS-CoV strains used for neutralization and challenge experiments, as well as vaccine-induced neutralizing-antibody titers, are described in parentheses.

Name	Functionality and Antigenicity	Immunogenicity in Induction of Antibody Response	Immunogenicity in Induction of Cellular Immune Response	Protective Immunity	Ref.
RBD-[SSG]-FR and RBD-FR nanoparticles	Bind to DPP4 receptor; antisera block RBD-hDPP4 binding	Induce MERS-CoV RBD-specific antibodies (IgG, IgG1, IgG2a, IgG2b, IgA) in mice	Elicit MERS-CoV RBD-specific T-cell responses (IFN-γ, TNF-α) in mouse splenocytes	N/A	[89]
sVLP (spherical virus-like particle)	N/A	Induces MERS-CoV RBD-specific antibodies (IgG) in mice, neutralizing pseudotyped MERS-CoV (1:320)	Elicits MERS-CoV RBD-specific cellular immune response (IFN-γ, IL-2, IL-4) in mouse splenocytes	N/A	[90]
rRBD (recombinant RBD)	N/A	Induces MERS-CoV RBD-specific antibodies (IgG, IgG1, IgG2a) in mice or NHPs, neutralizing pseudotyped (1:800 to 1:1,600) and live (EMC2012: 1:269 to 1:363) MERS-CoV	Elicits MERS-CoV RBD-specific cellular immune response (TNF-α, IFN-γ, IL-2, IL-4, IL-6) in mouse splenocytes or monkey PBMCs	Partially protects NHPs from MERS-CoV (EMC2012: 6.5 × 10^7^ TCID_50_) infection with alleviated pneumonia and decreased viral load	[88,91]
RBD (S377-588)-Fc	Binds strongly to soluble and cell-associated hDPP4 or cDPP4 receptors and MERS-CoV RBD-specific neutralizing mAbs (Mersmab1, m336, m337, m338)	Induces MERS-CoV S1-specific antibodies (IgG, IgG1, IgG2a) in mice and rabbits, cross-neutralizing 17 pseudotyped (>1:10^4^), 2 live (EMC2012, London1-2012: ≥1:10^3^) MERS-CoV, and 5 mAb escape mutants (>1:10^4^)	Elicits MERS-CoV S1-specific cellular immune responses (IFN-γ, IL-2) in mouse splenocytes	Protects Ad5/hDPP4-transduced BALB/c mice and hDPP4-Tg mice (67% survival rate) from challenge by MERS-CoV (EMC2012: 10^5^ PFU for BALB/c; 10^3–^10^4^ TCID_50_ for Tg), without toxicity or immune enhancement	[92,95,96,97,98,99]
2012-RBD 2013-RBD 2014-RBD 2015-RBD Camel-RBD	Bind strongly to hDPP4 and cDPP4 receptors and MERS-CoV RBD-specific mAbs (Mersmab1, m336, m337, m338) with high affinity	Induce MERS-CoV S1-specific antibodies (IgG, IgG1, IgG2a) in mice, potently cross-neutralizing 17 pseudotyped (≥1:10^4^), 2 live (EMC2012, London1-2012: >1:10^2^) MERS-CoV, and 5 mAb escape mutants (≥1:10^4^)	N/A	N/A	[100]
RBD-Fd	Binds strongly to soluble and cell-associated hDPP4 receptors and MERS-CoV RBD-specific mAbs (Mersmab1, m336, m337, m338)	Induces robust and long-term MERS-CoV S1-specific antibodies (IgG, IgG1, IgG2a) in mice, neutralizing at least 9 pseudotyped (>1:10^4^) and live (EMC2012: >1:10^3^) MERS-CoV	N/A	Protects hDPP4-Tg mice (83% survival rate) from lethal MERS-CoV (EMC2012: 10^4^ TCID_50_) challenge	[94]
RBD (T579N)	Binds strongly to soluble and cell-associated hDPP4 receptors and MERS-CoV RBD-specific mAbs (hHS-1, m336, m337, m338)	Induces highly potent neutralizing antibodies in mice against live MERS-CoV (EMC2012: >1:3 × 10^3^)	N/A	Significantly enhances efficacy in fully protecting hDPP4-Tg mice (100% survival rate) from lethal MERS-CoV (EMC2012: 10^4^ TCID_50_) challenge	[93]

Note: Ad5, adenovirus 5; DPP4, dipeptidyl peptidase 4; cDPP4, camel DPP4; hDPP4, human DPP4; hDPP4-Tg, hDPP4-transgenic; Fd, foldon; FR, ferritin; mAb, monoclonal antibody; N/A, not available or not applicable; NHP, non-human primate; PBMCs, peripheral blood mononuclear cells; PFU, plaque-forming unit; RBD, receptor-binding domain; S, spike; TCID_50_, median tissue-culture infectious dose.

**Table 2:** Therapeutic antibodies targeting MERS-CoV RBD. Live MERS-CoV strains used for neutralization and challenge experiments are indicated in parentheses.

Name	Source	Binding MERS-CoV RBD	Structure Available	In vitro Anti-MERS-CoV Activity	In vivo Protection	Ref.
MERS-GD27 MERS-GD33 mAbs	Human	*K*_d_: 0.775 nM (for MERS-GD27) and 0.575 nM (for MERS-GD33). Recognize RBD residues L506, D510, E513, W535, E536, D539, E540, W553 (for MERS-GD27), R511, and A556 (for MERS-GD33)	Yes, crystal structure for the Fab–RBD complex	IC_50_: 0.001 µg/mL against pseudotyped MERS-CoV; 0.001 µg/mL against live MERS-CoV; both mAbs have synergistic effect against pseudotyped MERS-CoV with reduced IC_50_ by 0.499-fold (for MERS-GD27) or 6.05-fold (for MERS-GD33) vs individual mAbs	MERS-GD27 prophylactically and therapeutically protects hDPP4-Tg mice against MERS-CoV (EMC2012: 3 LD_50_) with 60% and 40% survival rates, respectively	[112,113]
MCA1 mAb	Human	Recognizes RBD residues D510, W535, E536, D539, Y540, R542, and Q544	Yes, crystal structure for the Fab–RBD complex	IC_50_: 0.39 µg/mL against live MERS-CoV (EMC2012)	Prophylactically and therapeutically (5–20 mg/kg) inhibits MERS-CoV (EMC2012: 5 × 10^6^ TCID_50_) replication in common marmosets, improving clinical outcomes and reducing lung disease and viral replication	[114]
JC57-14 mAb	Macaque	Recognizes RBD residues W535, E536, D539, Y540, and R542	Yes, crystal structure for the Fab–RBD complex	IC_50_: 0.0084 µg/mL against pseudotyped MERS-CoV and 0.07 µg/mL against live MERS-CoV (EMC2012), cross-neutralizing 8 pseudotyped MERS-CoVs	N/A	[102]
CDC2-C2 mAb	Human	Recognizes RBD residues F506, D509, W535, E536, D539, Y540, and R542	Yes, crystal structure for the Fab–RBD complex	IC_50_: 0.0057 µg/mL against pseudotyped MERS-CoV and 0.058 µg/mL against live MERS-CoV (EMC2012), cross-neutralizing 10 pseudotyped MERS-CoVs	Prophylactically (20 mg/kg) protects hDPP4-Tg mice against MERS-CoV (EMC2012: 10^6^ TCID_50_) in lungs with 100% survival rate	[102]
MERS-4 mAb	Human	Recognizes RBD residues L507, L545, S546, P547, G549; binds RBD from outside of the RBD DPP4-binding interface	Yes, crystal structure for the Fab–RBD complex	Has synergistic neutralization effect with MERS-27, m336, and 5F9 mAbs against pseudotyped MERS-CoV, with the reduction of IC_50_ by 2.6-fold (for MERS-4 + m336) and 15.21-fold (for MERS-4 + 5F9)	N/A	[115]
VHH-83 HCAb-83 VHHs	Dromedary	*K*_d_: 0.1 nM (for VHH-83) and 2.5 pM (for HCAb-83). Recognizes RBD residue D539	N/A	PRNT_50_: 0.0012–0.0014 µg/mL against live MERS-CoV (EMC2012)	HCAb-83 (200 µg) prophylactically protects hDPP4-Tg mice (K18) against MERS-CoV (EMC2012: 10^5^ PFU) in lungs with 100% survival rate	[116]
NbMS10 NbMS10-Fc VHHs	Llama	*K*_d_: 0.87 nM (for NbMS10) and 0.35 nM (for NbMS10-Fc). Recognize RBD residue D539	N/A	IC_50_: 0.003–0.979 µg/ml (for NbMS10) and 0.003–0.067 µg/ml (for NbMS10-Fc) in cross-neutralizing ≥11 pseudotyped MERS-CoVs	NbMS10-Fc (10 mg/kg) prophylactically and therapeutically protects hDPP4-Tg mice against MERS-CoV (EMC2012, 10^5.3^ TCID_50_) with 100% survival rate	[117]

Note: DPP4, dipeptidyl peptidase 4; Fab, antigen-binding fragment; hDPP4-Tg, human DPP4-transgenic; IC_50_, half-maximal inhibitory concentration; *K*_d_: antibody binding affinity; LD_50_, 50% lethal dose; N/A, not available or not applicable; PRNT_50_, 50% plaque-reduction neutralization titer; RBD, receptor-binding domain; TCID_50_, median tissue-culture infectious dose; VHH, single-domain antibody fragment.
